# Virus-like Particle Vaccines and Platforms for Vaccine Development

**DOI:** 10.3390/v15051109

**Published:** 2023-05-02

**Authors:** Milad Kheirvari, Hong Liu, Ebenezer Tumban

**Affiliations:** School of Veterinary Medicine, Texas Tech University, Amarillo, TX 79106, USA

**Keywords:** virus-like particles, chimeric VLPs, vaccines, immunogenicity, efficacy

## Abstract

Virus-like particles (VLPs) have gained a lot of interest within the past two decades. The use of VLP-based vaccines to protect against three infectious agents—hepatitis B virus, human papillomavirus, and hepatitis E virus—has been approved; they are very efficacious and offer long-lasting immune responses. Besides these, VLPs from other viral infectious agents (that infect humans, animals, plants, and bacteria) are under development. These VLPs, especially those from human and animal viruses, serve as stand-alone vaccines to protect against viruses from which the VLPs were derived. Additionally, VLPs, including those derived from plant and bacterial viruses, serve as platforms upon which to display foreign peptide antigens from other infectious agents or metabolic diseases such as cancer, i.e., they can be used to develop chimeric VLPs. The goal of chimeric VLPs is to enhance the immunogenicity of foreign peptides displayed on VLPs and not necessarily the platforms. This review provides a summary of VLP vaccines for human and veterinary use that have been approved and those that are under development. Furthermore, this review summarizes chimeric VLP vaccines that have been developed and tested in pre-clinical studies. Finally, the review concludes with a snapshot of the advantages of VLP-based vaccines such as hybrid/mosaic VLPs over conventional vaccine approaches such as live-attenuated and inactivated vaccines.

## 1. Introduction

**Figure 1:**
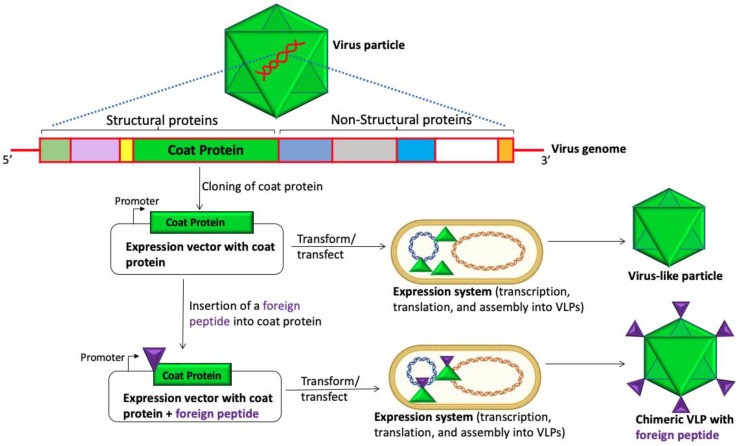
A schematic illustrating the generation of VLPs and chimeric VLPs. A coat protein from a virus is cloned to an expression vector. The vector with the coat protein can also be used to insert a foreign peptide into the coat protein (bottom). Each vector is then transformed or transfected to an expression system where the proteins are expressed and assembled into VLPs.

Viruses infect a wide range of organisms, ranging from humans, plants, animals, birds, and insects to microorganisms (e.g., eukaryotes and prokaryotes). One unique feature of viruses is that their structural proteins, envelope proteins, or capsid proteins, along with other structural proteins, can, either independently or collectively, spontaneously self-assemble to form virus-like particles (VLPs) without the viral genome. Thus, any virus can be utilized to develop VLPs; VLPs can be developed by cloning the structural genes that code for the proteins of a virus of interest into an expression vector (Figure 1). The expression vector depends on the expression system in which the protein(s) will be expressed and, sometimes, the protein is codon-optimized if the expression system (including that of mammalian, insects, and bacteria) is different to the cells which the virus of interest normally infect. The vector harboring the DNA of the structural protein(s) is then transfected/transformed into cells of interest, where the DNA is transcribed and translated. Translated protein folds and assembles to form VLPs [1]. VLPs have many applications in biomedical sciences, such as: (i) therapy—the delivery of drugs/cargo to specific cancer cells; (ii) in vivo imaging—VLPs loaded with fluorophores; (iii) diagnostic tests—utilizing armored RNA as positive controls for infectious diseases; iv) vaccine development [2,3,4]. This review focuses only on the latter: the application of VLPs in vaccine development.

**Figure 2:**
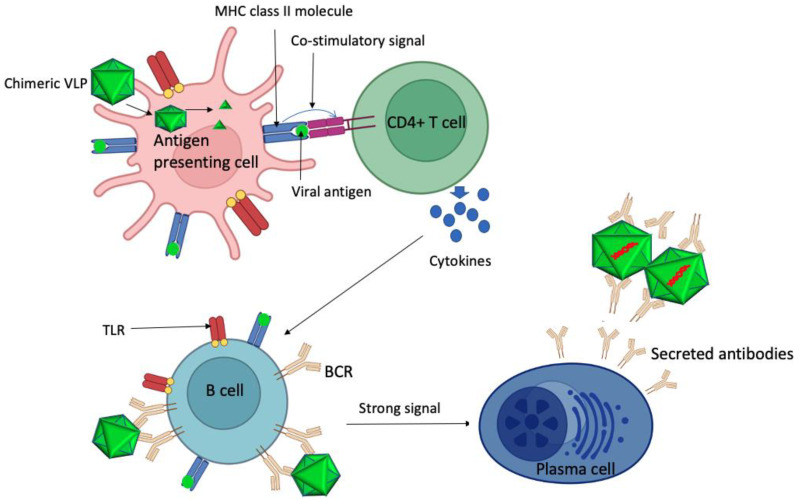
The activation of immune responses by VLPs. Antigen-presenting cells phagocytose and process VLPs into fragments, which are presented to T-helper cells with the help of MHC class II (tope image). This leads to the activation of T-helper cells, which secrete cytokines that activate B-cells (below). B-cells are also activated by the cross-linking of B-cell receptors (BCRs) by VLPs. Activated B-cells divide and differentiate into plasma cells and memory cells (not shown here). Plasma cells secrete antibodies into the body, which neutralize the virus of interest from which the VLPs were derived. The figure is adopted from [5].

VLPs have many features, unlike conventional vaccines, which make them very attractive platforms for vaccine design. They mimic the viruses from which the VLPs are derived in terms of size (20–200 nm) [5], geometry (i.e., icosahedral structures with multivalent epitopes) [5,6,7,8], and the ability to activate T-helper cells. VLPs naturally encode T-helper cell epitopes, which are presented to T-helper cells by antigen-presenting cells (APCs) in association with major histocompatibility complex (MHC) class II. The presentation of the epitopes in addition to the co-stimulatory molecules from APCs leads to the activation of T-helper cells. This activates T-helper cells’ secret cytokines that activate other immune cells, such as macrophages, B-cells, and T-cells (Figure 2) [6]. VLPs can also be engineered to encapsidate endogenous adjuvants such as single-stranded (ss)RNA to stimulate innate immune responses as follows [5,9,10,11]: ssRNA binds to Toll-like receptors TLR7 and TLR8, which are located in the intracellular compartments (e.g., the endosome) of immune cells. The binding of ssRNAs to TLR7 and TLR8 leads to the activation of the receptors, which send signals downstream, leading to the expression/secretion of cytokines (e.g., interferon α, interferon γ, TNFα, IL-1β, IL-6, and IL-12). Secreted cytokines activate innate immune cells, as well as the cells of the adaptive immune system which control the invasion of pathogens [12,13,14,15]. This feature also enhances the immunogenicity of VLPs even at lower doses [16,17].

Moreover, VLPs are considered safe, since they do not contain the viral genome, and therefore, they cannot replicate. It is worth mentioning that, like any vaccine, VLPs can cause side effects such as pain and swelling at the injection site. Hence, it is no surprise that VLPs have gained considerable attention over the past two decades as an attractive platform for vaccine design. While VLPs derived from human and animal viruses are developed to protect humans and animals against viruses from which the VLPs are derived, VLPs from other organisms (including plants and prokaryotic microorganisms) are developed to display heterologous antigens from human and animal viruses, i.e., to develop chimeric VLPs (Figure 1, bottom). The goal of chimeric VLPs is to elicit immune responses against heterologous antigens displayed on the platform and not necessarily the platform. It is worth mentioning that VLPs derived from human or animal viruses can also be used to develop chimeric VLPs, whereby a heterologous antigen displayed on the VLP is derived from other human viruses (Figure 1, bottom).

## 2. VLP Vaccines Derived from Human Viruses

VLPs have been developed (while others remain under development) using viruses that infect humans (Table 1). In fact, VLP-based vaccines have been approved to protect against three human viral infections, namely, hepatitis B virus (HBV), human papillomaviruses (HPV), and hepatitis E (HEV). Among these, at least nine HBV VLP-based vaccines have been approved globally, and these include Engerix-B, Recombivax HB, Euvax B, Hepavax-Gene, GenHevac B, GenVac B, and Heberbiovac HB (this is reviewed in [1,18,19]). The vaccines are based on the HBV surface antigen; some of the vaccines; for example, Engerix-B and Recombivax HB, have been in use since the mid-1980s, and they offer cross-protection against other HBV genotypes, such as A and C [20]. Although the vaccines cross-protect against other genotypes, several vaccine-escape viral mutants have been reported for the vaccines (reviewed in [21]). Regardless of this, HBV protective antibodies last up to 30 years in some individuals [22,23]. Age at the time of vaccination seems to be associated with HBV antibody responses. For example, individuals vaccinated at an average age of 36 years developed fewer anti-HBV antibodies compared to those who were vaccinated at an average age of 32 years. However, a single boost enhanced antibody levels in 94% of individuals who had low levels of antibodies [22,23].

Three VLP-based vaccines—Gardasil-9, Gardasil-4, and Cervarix—have been approved around the world to protect against human papillomaviruses [24,25,26,27,28,29]. These VLPs are derived from the major capsid protein L1; they offer protection against two to seven HPV types—HPV16, 18, 31, 33, 45, 52, and 58—and these are associated with 70–90% of cervical cancer cases. They also offer protection against two HPV types—HPV6 and 11—associated with ~90% of genital warts [29]. Immune responses to Cervarix and Gardasil-4 last at least 13 years [30,31,32], while those for Gardasil-9 last at least 6 years [33]. The Cervarix vaccine offers 21–64% cross-protection against HPV35, 31, 33, 45, and 58 after 11 years. On the other hand, 90% of individuals vaccinated with Gardasil-4 had antibodies against heterologous HPV types (HPV6, 11, and 16) and 52% had antibodies against HPV18 after 14 years [30,31,32]. It is worth mentioning that longevity studies for all three vaccines are ongoing. Two other HPV vaccines with the potential to offer broader protections are in clinical trials; an 11-valent candidate vaccine with undisclosed HPV types [34] and a 14-valent candidate vaccine [35] are in phase III and phase I clinical trials, respectively. The 14-valent candidate vaccine is expected to protect against HPV6, 11, 16, 18, 31, 33, 35, 39, 45, 51, 52, 56, 58, and 59.

One VLP-based vaccine (Hecolin) used to protect against HEV is approved in China; clinical trials to assess its efficacy in other countries—Bangladesh and the U.S.—are ongoing [36,37]. The vaccine is based on amino acids 368–606, derived from the open reading frame 2 of the capsid protein of HEV genotype 1. Vaccine efficacy after 12 months and three doses in 16–64-year-old individuals is 100% [38]. In the elderly, i.e., >65 years old, 97.3% of individuals vaccinated with Hecolin seroconvert 1 month after the third dose at month 7 [39]. Moreover, 87% of the vaccinees had detectable antibodies that lasted for at least 4.5 years [40]. The vaccine protects against genotypes 1, 2, and 4 and is expected to cross-protect to a lesser extent against genotype 3 [41,42]. 

In addition to the approved VLP-based vaccines above, several candidate VLP-based vaccines are under development. An excellent new book by Pumpens and Pusko provides a comprehensive summary of candidate VLP-based vaccines that have been developed from various viruses [1]. VLPs that have been developed and tested include influenza viruses, SARS-CoV-2, human immunodeficiency virus, and the Zika virus. Table 1 summarizes some of the VLPs developed from human viruses in pre-clinical and clinal trails. The VLPs are derived from either the envelope protein, the capsid protein, or both and are expressed using various expression systems, such as mammalian cells, insect cells, yeast cells, and bacterial cells, as well as transgenic plants. The efficacy of some of these VLPs is comparable to those of approved vaccines developed using conventional approaches. For instance, immunization with hybrid VLPs developed using structural proteins from two influenza A subtypes—H1N1 and H3N2—is as immunogenic as an approved inactivate influenza virus vaccine (Vaxigrip; H2N3); IgG responses had a normalized median fluorescence intensity of >10^7^ [43]. Similarly, the immunization of mice with VLPs derived from the Zika virus or Ebola virus offers a survival rate of up to 100%, unlike control mice, where there was no survival [44,45].

VLPs derived from human viruses have also been used to develop candidate vaccines against other human viruses. For example, VLPs derived from HBV have been used to develop candidate vaccines against *Helicobacter pylori* (*H. pylori*) bacteria, hepatitis C virus (HCV), HPV-associated cancers, and four serotypes of dengue virus (Table 2). In preclinical studies, mice immunized with chimeric HBV VLPs displaying peptides derived from *H. pylori* had a reduced bacterial burden [46]. In other studies, sera from mice immunized with HBV VLPs displaying peptides from HCV and dengue viruses neutralized and protected mice from HCV and dengue virus infection, respectively [47,48]. Additionally, HBV VLPs displaying peptides from HPV16 E7 suppress tumors in a mouse model for HPV-associated cancer [49].

**Table 1:** VLPs derived from human viruses.

Name of Virus	Structural Protein Used to Make VLPs	Source of Structural Protein (Capsid/Envelope)	Expression System	Immune Responses	References
Influenza A subtypes (H1N1 and H3N2)	Hemagglutinin (HA), matrix protein 1 (M1), and neuraminidase (NA)	Envelope	Mammalian cell lines (Chinese hamster ovary cells—CHO-K1, vero cells, human embryonic kidney—HEK 293T cells)	Mice immunized with the hybrid VLPs elicited antibody titers against A/Hong Kong (H3N2) strain that were similar to those of an approved inactivated vaccine (Vaxigrip)	[43]
			*Spodoptera frugiperda* (Sf)9 insect cells	Ferrets immunized with the VLPs were protected against Influenza virus A H3N2	[50]
Influenza A virus (H7N9)	HA, M1, NA		Sf9 insect cells	Hemagglutination inhibition antibody titers against H7N9 were from 1:80 to 1:173	[51]
Influenza B/Shanghai/361/2002				Hemagglutination inhibition antibody titer against the strain was 1:1280	[50]
Influenza viruses	Headless HA gene with an extracellular region of matrix protein 2 gene insertion (from human, avian, and swine influenza), nucleoprotein, and M1 from H5N1	Envelope	Sf9 insect cells	Mice immunized with chimeric VLPs were protected against homologous (H5N1) and heterologous influenza viruses (H1N1, H3N2, or H7N7); infections were reduced by 4.6–6.7-fold.	[52]
HPV	Major capsid protein (L1)	Capsid	*Saccharomyces cerevisiae* (*S. cerevisiae*)	Gardasil-9 vaccine contains VLPs from 9 HPV types; protects against HPV associated with 90% of cervical cancer and 90% of genital warts	[53]
			*Trichoplusia ni* insect cells	Cervarix vaccine contains VLPs from HPV16 and HPV18; protects against these two HPV types (associated with ~70% of cervical cancer); it also cross-protects against other HPV types	[54,55]
HPV (type 6 and 11)	L1	Capsid	*E. coli*	Neutralizing antibody titers (100–1000 in monkeys) against HPV6 and HPV11 were similar to those of Gardasil-4	[56]
HBV	HBV small surface antigen (HBsAgS)	Envelope	Yeast cells [*S. cerevisiae*, *Pichia pastoris* (*P. pastoris*), *Hansenula polymorpha*]	Immune responses cross-protect against different serotypes and last up to 30 years (Engerix-B, Recombivax HB vaccines)	[20,21,22,23]
	HBsAgS and middle protein		Mammalian cells (CHO)	94% of vaccines seroconvert and 84% were seroprotected (GenHevac B vaccine)	[57]
HEV	Capsid protein	Capsid	*E. coli*	Hecolin vaccine (HEV 239) has efficacy of 97–100%. It cross-protect against other genotypes. Immunity lasts for at least 4.5 years.	[38,39,40,41,42]
Human immunodeficiency virus (HIV) type 1 (HIV 1)	Envelope and Gag	Envelope and capsid	HEK 293T cells	Immunization with the VLPs, without any adjuvant, elicited neutralizing antibodies and cytotoxic T-cell responses in mice.	[58]
	Glycoprotein (gp) 120	Envelope	*Sf*9 insect cells	Sera derived from mice immunized with VLPs neutralized (at 1:10–1:80) homologous and heterologous isolates of HIV	[59,60,61]
Human noroviruses (genotype GI.1 and consensus GII.4) Human norovirus (consensus GII.4)	Virus protein (VP)1	Capsid	*Sf*9 insect cells	Seven days post-immunization, IgA and IgG antibody secreting cells in humans increased by more than 4-fold for genotype GI.1 and consensus GII.4 viruses	[62]
	VP1	Capsid	*P. pastoris*	Sera and fecal antibodies derived from mice immunized with VLPs block binding of VLPs to receptors	[63]
Parvovirus B19	VP1, VP2	Capsid	*S. cerevisiae*	VLPs elicited high antibody responses (>4 logs) in a mouse model for sickle cell disease; responses persisted for >80 days.	[64]
	VP1, VP2	Capsid	*Sf*9 insect cells	Geometric mean neutralizing antibody titers in humans ranged from 6.45–20.29; reactogenicity was reported in a lot (up to 73%) of participants	[65]
** Chikungunya virus (CHIKV)	Capsid (envelope) 1, E2, E3, 6K	Capsid and Envelope	*P. pastoris*	Passive transfer of antibody to neonatal mice offered protection from CHIKV infection	[66,67,68,69]
			HEK 293T cells	IgG from monkey immunized VLPs protected mice from dying following lethal infection with CHIKV	
			*SF*21 cells	Mice vaccinated with VLPs were protected from viremia/arthritis following infection with CHIKV	
Coxsackievirus A16 (CA16)	VP1, VP3, and VP0 (VP2 and VP4)	Capsid	*S. cerevisiae*	Passive transfer of anti-CA16 VLP sera to neonatal mice protected mice against lethal CA16 challenge	[70]
Enterovirus 71 (EV71)	VP0, VP1, VP3	Capsid	*S. cerevisiae*	90–100% of neonatal mice infected with a mixture of EV71 VLP-derived sera and EV71 were protected from infection (i.e., did not die)	[71,72]
Nipah virus	-Glycoprotein, Matrix protein, Fusion protein	Envelope	HEK 293T cells	All ferrets immunized with Nipah virus VLPs survived infection with Nipah virus compared to control animals (40–75%)	[73]
Rotavirus	VP2, VP6, VP7	Capsid	Transgenic tobacco plants	Mice orally immunized with VLPs (made up of VP2/6/7) elicited serum IgG and fecal IgA antibodies; serum antibody levels were comparable to those of an attenuated rotavirus vaccine.	[74]
SARS-CoV-2	Spike	Envelope	*Nicotiana benthamiana* tobacco plant	Neutralizing antibody titers in immunized individuals were greater than those in individuals recovering from COVID-19.	[75]
Dengue virus 2	Envelope protein and 5′ pre-membrane signal peptide	Envelope	*P. pastoris*	AG129 mice immunized with VLPs elicited neutralizing antibodies (titers > 1200) that protected them against lethal challenge with homologous dengue virus 2.	[76]
Zika virus	Envelope protein and pre-membrane	Envelope	HEK 293T cells	100% of AG129 mice immunized with 10 μg of VLPs survived infection with Zika virus compared to control mice (no survival) after day 21.	[44]
Japanese encephalitis virus (JEV)	Envelope protein and pre-membrane	Envelope	Lepidoptera mosquito cells	Vaccinated mice elicited a balanced immune response (Th1/Th2) and neutralized both JEV genotypes I and III (neutralizing antibody titers 10–320).	[77]
Ebola virus	Glycoprotein (GP) and matrix protein	Envelope	HEK 293T cells	30% of mice immunized with VLPs (without adjuvant) survived following challenge with a mouse-adapted Ebola virus strain; 100% of mice survived infection when they were immunized with the VLPs in the presence of GLA-SE or GLA-AF adjuvants.	[45]
Ebola virus Sudan strain	GP and matrix protein	Envelope	*Sf*9 insect cells	Sera from horses immunized with VLPs blocked infection with HIV pseudovirus expressing Ebola Sudan glycoproteins	[78]

** Completed phase I clinical trial [69].

## 3. VLP Candidate Vaccines Derived from Veterinary Viruses

VLPs derived from viruses that infect animals and fish have also been used to develop vaccines against viruses that affect various animals, including pigs, rabbits, sheep, horses, chickens, and fish(Table 3). For instance, VLPs derived from porcine circovirus-2 (PCV-2) elicit better antibody responses in mice, superior to those of a commercial subunit vaccine [86]. PCV-2 is associated with postweaning multisystem wasting syndrome in young pigs. In sheep, immunization with VLPs derived from bluetongue virus offers the same level of protection as immunization as a live-attenuated commercial vaccine (monovalent BTV-8) [87]. In foals, immunization with chimeric VLPs derived from three African horse sickness viruses—AHSV-6, AHSV-3, and AHSV-1—elicited neutralizing antibodies against these viruses, albeit at low levels [88]. In chickens, immunization with mosaic VLPs derived from different subtypes/strains of influenza A or immunization with VLPs derived from infectious bursal disease virus offered better protection than commercial inactivated vaccines (e.g., H6N2 for influenza virus) [89,90,91] (Table 3). Meanwhile, in fish, immunization with VLPs derived from red-spotted grouper nervous necrosis virus (RGNNV) lowered the mortality rate to ~3.3%, compared to immunization with a commercially inactivated vaccine, OceanTect viral nervous necrosis, and the control group (which had mortality rates of 10% and ~79%, respectively) [92]. Additionally, the immunization of fish with VLPs derived from Atlantic cod nervous necrosis virus lowered the mortality rate to 14%, compared to immunization in the control group, where the mortality rate was lowered to only ~80% [93].

VLPs derived from animal viruses have also been used as platforms to develop candidate vaccines against other viral infections (Table 4). For example, VLPs from canine parvovirus have been used to display peptide antigens from Middle East respiratory syndrome coronavirus (MERS-CoV) and the VLPs elicited a balanced immune response in mice; sera from immunized mice neutralized pseudo-MERS-CoV [94]. Furthermore, immunization with VLPs derived from Newcastle disease virus displaying a Brucella antigen—BCSP31—offered protection against a virulent strain (16M) of *Brucella melitensis*; the protection level was similar to that of a commercial live-attenuated vaccine: *Brucella melitensis* strain M5 [95]; *Brucella melitensis* is a zoonotic disease associated with brucellosis.

**Table 3:** VLPs derived from animal and fish viruses.

Name of Virus	Structural Protein Used to Make VLPs	Source of Structural Protein (Capsid/Envelope)	Expression System	Immune Responses	References
PCV-2	Capsid protein	Capsid	*Nicotiana benthamiana*	Mice immunized with PCV VLPs elicited antibodies 42 days after immunization. Antibody levels were higher than those elicited by a commercial subunit vaccine (Ingelvac CircoFLEX^®^).	[86]
Porcine parvovirus (PPV)	VP2	Capsid	*E. coli*	Vaccinated mice and pigs generated neutralized antibodies; antibodies significantly reduced PPV content in the spleen of pigs 14 days after PPV challenge.	[96]
Rabbit hemorrhagic disease virus (RHDV)	VP60 from two genotypes (RHDV GI.1- and RHDV GI.2)	Capsid	*Tricholusia ni* insect pupae	Hybrid VLPs elicited antibodies that protected rabbits against lethal challenge with the 2 RHDV genotypes, (RHDV. GI.1 and GI.2). Immunization with 40 µg offered 100% protection compared to immunization with 20 µg (80% protection).	[97]
Bluetongue virus	VP2, VP3, VP5 and VP7 from serotype 8	Capsid	*Nicotiana benthamiana*	Sheep immunized with VLPs had the same efficacy of protection as the live-attenuated commercial vaccine; no clinical signs of disease were observed.	[87]
AHSV	VP2, VP3, VP5, and VP7 from serotype 5	Capsid	*Nicotiana benthamiana*	Sera from guinea pigs immunized with VLPs neutralized serotypes 5 and 8 (to a lesser degree). No cross-neutralization of serotype 4.	[98,99]
	VP2, VP3, VP5 and VP7 from serotype 1; VP2, VP5, from serotype 7; VP2, from serotype 6; VP5 from serotype 3	Capsid	*Nicotiana benthamian*	Single, double, and triple chimeric VLPs were developed; anti-VP7 specific responses were detected in foals immunized with triple chimeric VLP (AHSV-6/AHSV-3/AHSV-1). However, single AHSV-6 VLPs elicited a weak neutralizing humoral immune response against homologous AHSV virus. Low neutralization levels were also observed with a control live-attenuated AHSV-6 vaccine.	[88]
Goose hemorrhagic polyomavirus (GHPV)	VP1 with or without VP2	Capsid	*Sf*9 insect cells and *S. cerevisiae*	The VLPs expressed by yeast were of smaller size. VLPs (as a diagnostic antigen) detected GHPV-specific antibodies in up to 85.7% of geese sera with hemorrhagic nephritis and enteritis.	[100]
Canine influenza virus (CIV) H3N2	M1 and hemagglutinin proteins	Envelope	*Sf*9 insect cells	Dogs vaccinated with the VLPs and later challenged with CIV H3N2 did not show clinical signs of respiratory disease, unlike control dogs.	[101]
Influenza A virus	HA protein from (A/chicken/South Africa/N2826/2016 (H6N2)) and M2 protein from strain A/New Caledonia/20/1999 (H1N1)	Envelope	*Nicotiana benthamiana*	A total of 100% of chickens immunized with the mosaic VLPs did not shed virus via the respiratory tract (at day 21) following a challenge with strain A/chicken/South Africa/H44954/2016 (H6N2): 58% of chickens immunized with a commercial inactivated H6N2 vaccine shed virus, as opposed to 36% unimmunized.	[89]
Influenza A virus	Hemagglutinin antigen from H5N1, H7N3 and H9N2 viruses. neuraminidase 1 antigen from influenza H5N1 and gag protein from a retrovirus	Envelope	*Sf*9 insect cells	Chickens immunized with mosaic VLPs and later challenged with H5N2 and H7N3 viruses survived while all control unimmunized birds died.	[90]
Rabies virus	Glycoprotein	Envelope	HEK 293	Antibody titers were >4 log_10_ and were similar to those of two licensed inactivated rabies vaccines (for humans and animals). A 0.3 μg dose elicited similar antibody titers. Elicited antibodies neutralized a pseudotyped lentivirus (expressing rabies virus G protein).	[17,102]
Foot-and-mouth disease virus	VP1 from serotype O and VP2, VP3, and VP4 from serotype A	Capsid	*Sf*9 insect cells	Guinea pigs immunized with the mosaic VLPs elicited both humoral and cellular immune responses; the protective efficacy of the mosaic VLPs against serotype O virus was 80% compared to 0% in the control group.	[103]
Infectious bursal disease virus (IBDV)	VP2 protein	Capsid	*P. pastoris*	All chickens immunized with IBDV VLPs survived after challenge with the virus; 10% of chickens immunized with a commercial inactivated vaccine died; 80% of control chickens died.	[91]
RGNNV	Capsid protein	Capsid	*Nicotiana tabacum cv. Xanthi*	Cumulative mortality (within 14 days) in fish vaccinated with RGNNV-VLPs was 3.3% compared with 10% and 60–66.7% mortality in fish immunized with commercial inactivated vaccine and control plant extract, respectively.	[92]
			*S. cerevisiae*	Mice immunized with the VLPs elicited saturated IgG antibody titers (5.8 log_10_).	[104]
Piscine myocarditis virus	ORF1	Capsid	*Nicotiana benthamiana*	VLPs elicited an innate immune response in fish, which was associated with reduced viral replication in the heart, spleen, and kidney of salmon, and reduced inflammatory lesions in cardiomyocytes.	[105]
Atlantic cod nervous necrosis virus	Capsid protein	Capsid	*Nicotiana benthamiana* and tobacco BY-2 cells	VLPs significantly lowered the mortality in vaccinated groups compared to control group; vaccinated fish showed relatively higher percent survival (from 63.6 to 86.5%) compared to the control group (~20.8%).	[93]

## 4. VLP Candidate Vaccines Derived from Plant and Bacterial Viruses

VLPs have been developed not only from human and animal viruses, but also from plant viruses and bacteria viruses. Plant-derived VLPs have been developed using various viruses such as alfalfa mosaic virus (AMV), physalis mottle virus (PhMV), potato virus Y (PVY), cucumber mosaic virus, and malva mosaic virus (MaMV) (Table 5). VLPs from AMVs displaying an antigen (Pfs25 protein) from *Plasmodium falciparum* have been shown to be safe and tolerable in a phase I clinical trial [110,111]. PhMV VLPs displaying HER2 peptide were found to slow tumor growth in mice and delay their death [112,113]. In addition to this, PVY VLPs displaying a cat allergen peptide—*Feline domesticus*—elicited high-antibody titers against the antigen [114]. Moreover, immunization with MaMV-displaying canine influenza virus H3N8 antigen protected mice from lethal challenge with homologous and heterologous mouse-adapted influenza virus strains, unlike control mice [115]. 

On the other side of viruses that infect bacteria (bacteriophages or phages), VLPs have been developed from bacteriophage AP205, MS2, PP7, Qβ, P22, etc. (Table 6). Immunization with MS2 and PP7 VLPs displaying HPV peptides protects mice from infection with 11 HPV pseudovirus types associated with cancer [116,117,118]. Bacteriophage VLPs have also been used in cancer research. Bacteriophage P22 VLPs displaying B and T cell epitopes from ovalbumin inhibit EG.7-OVA lymphoma cells in mice [119].

**Table 5:** VLPs derived from plant viruses (chimeric VLPs) displaying foreign antigens.

Name of Virus Used to Develop VLPs	Structural Antigen Used to Make VLPs	Capsid or Envelope Proteins	Foreign Antigen Displayed on VLP	Expression System	Immune Responses	References
** AMV	Capsid protein	Capsid	Pfs25 protein of Plasmodium falciparum	Nicotiana benthamiana tobacco plants	In a phase I study, VLPs were shown to be safe and tolerable; reactogenicity was also reported. IgG responses > 3 log_10_ were observed with a dose of 100 μg.	[110,111]
Cowpea chlorotic mottle virus (CCMV)	Capsid protein	Capsid	Tetanus toxin epitope	*E. coli*	Round-shaped CCMV_TT_-VLPs drained more efficiently to secondary lymphoid organs than rod-shaped CCMV_TT_-VLPs. Additionally, round-shaped CCMV_TT_-VLPs increased IgG and IgA antibody levels by 100-fold compared to rod-shaped CCMV_TT_-VLPs.	[120]
	Capsid protein	Capsid	S9 peptide from group B streptococcal type III capsular polysaccharide	*P. pastoris*	Immunization with VLPs displaying S9 peptide elicited a Th1 response against the peptide. However, immunization with the peptide conjugated to keyhole limpet hemocyanin elicited a Th2 response.	[121]
PhMV	Capsid protein	Capsid	HER2-derived CH401 epitope	*E. coli*	VLPs elicited a strong immune response. Chimeric VLPs slowed the growth of DDHER2 tumor cells in mice and also delayed death by more than 15 days compared to control mice that were immunized with just the CH401 epitope.	[112,113]
PVY	Capsid protein	Capsid	HBV preS1 epitope	*E. coli*	Chimeric VLPs elicited high-titer (1:8620) anti-HBV preS1 antibodies in mice without adjuvant.	[122]
			Cat allergen Feline domesticus (Fel d 1)	*E. coli*	All chimeric VLP vaccines elicited high antibody titers (up to 1:52,938) against Fel d 1 in mice. IgG1 was the dominant IgG subclass produced.	[114]
Cucumber mosaic virus	Capsid protein	Capsid	T-cell epitope derived from the tetanus toxin, dimeric murine IL-17A of psoriasis, cat allergen feline domesticus 1 (Fel d 1), and Aβ_1–6_ of Alzheimer β-amyloid	*E. coli*	Chimeric VLPs elicited antibodies that protected/reduced psoriatic disease and cat allergy. Antibodies from mice immunized with VLPs displaying Aβ_1–6_ reacted with plaques of Aβ (in brain sections from Alzheimer’s patients).	[123]
Papaya mosaic virus	Capsid protein	Capsid	HCV E2 epitope	*E. coli*	Chimeric VLPs elicited HCV E2 antibodies that lasted more than 4 months. A balanced T cell (Th1/Th2) immune response was observed.	[124]
Tobacco mosaic virus	Capsid protein	Capsid	L2 epitopes from cottontail rabbit papillomavirus and rabbit oral papillomavirus	*Nicotiana benthamiana* or *Nicotiana tabacum*	Rabbits immunized with the chimeric VLPs were protected from developing papillomas.	[125]
MaMV	Capsid protein	Capsid	M2e peptide from influenza virus A	*E. coli*	Mice immunized with the VLPs elicited antibodies that protected them from death following challenge with homologous mouse-adapted virus (H3N8 virus) or heterologous mouse-adapted virus (H1N1). Control mice died.	[115]

** Completed phase I clinical trial.

Overall, VLPs from viruses that infect humans, animals, plants, and bacteria have been used to develop vaccines/candidate vaccines against infectious agents, as well as metabolic diseases. Table 1, Table 2, Table 3, Table 4, Table 5 and Table 6 provide an overview of the different VLPs developed from various viruses and their potential applications.

## 5. Expert Review Commentary and Future Perspectives

VLP vaccines are proteins, and thus, they cannot be amplified in the body (i.e., transcribed and translated to make more copies) like other recombinant vaccines such as viral vector vaccines and mRNA vaccines. Nevertheless, VLP vaccines are better alternatives to the two recombinant vaccines. VLP vaccines do not need freezing conditions of −20 to −80 °C for transportation and storage, like mRNA vaccines. In addition to this, VLPs are immediately processed by APCs once they are injected into the body, unlike DNA and mRNA vaccines, whereby cells first need to transcribe (for DNA vaccines) and translate their mRNAs into proteins before they can be processed by APCs to be presented to the immune system.

VLP vaccines are also better alternatives to conventional vaccines (live-attenuated vaccines and inactivated vaccines) for several reasons: First, VLP vaccines do not replicate, and thus, they can be used for everyone, including women who are pregnant or people with a compromised immune system. Additionally, coat proteins—including other structural proteins—from viruses that have segmented genomes (e.g., influenza viruses, AHSV, and bluetongue virus) can also be used to develop hybrid/mosaic VLP-based vaccines without fear of genetic reassortment, like in live-attenuated vaccines. Viruses with segmented genomes have the ability to undergo genetic reassortment; unfortunately, a live-attenuated polyvalent vaccine derived from an AHSV led to the virulent reversion of AHSV type 1 and genomic reassortments with segments from AHSV types 1, 3, and 4, leading sporadic outbreaks of AHSV [128]. Second, VLPs closely mimic the structure of authentic viruses, unlike inactivated vaccines, whose structural proteins may be modified during inactivation, leading to compromised immunogenicity [129]. VLP vaccines, therefore, serve as a better alternative to inactivated vaccines. They are also immunogenic at lower doses [16,17]; in fact, studies show that immunization with 0.3 μg of a candidate rabies virus VLP vaccine elicits antibody titers that are comparable to immunization with 3 μg of the VLPs or with veterinary and human vaccines [17].

Although VLP vaccines are immunogenic at smaller doses, it is unclear whether smaller doses of VLPs can provide long-term protection against viruses with different genotypes such as HBV (which has at least nine genotypes [130]), HPV (more than 20 types associated with cancers [29]), or HEV (which has at least seven genotypes [131]). As mentioned above, immune responses to VLP-based vaccines offer cross-protection against different HBV, HPV, and HEV genotypes [20,29,41,42] but less protection against other genotypes or escape mutants (e.g., HBV) [21,41,42]. These vaccines are given at doses of at least 10 μg/immunization (for example, Engerix-B for HBV [132]); therefore, reducing the dose/immunization may not enhance cross-protection. We believe that increasing the concentration of VLPs (antigens in general) or the number of booster doses may enhance cross-protection. For example, studies have shown that cross-protection against an influenza virus subtype and mice survival following challenge are enhanced at higher doses of antigen, for example, 90 μg, compared to 10 μg [133]. Similarly, immunization with a high dose (10^6^ plaque forming units) of an ASFV vaccine offers complete cross-protection against an ASFV strain compared to partial cross-protection following immunization with half the dose [134].

VLP-based vaccines against viruses of interest should therefore elicit cross-protective immunity against all genotypes of the virus of interest to ensure global efficacy, given the fact that the distribution of genotypes vary from one geographical region to another. For example, HBV genotypes vary from one continent to another, with genotype A being more prevalent in North America, South America, Africa, and Europe, while genotypes B and C are prevalent in Southeast and East Asia. Genotypes D is prevalent in western/central/southern Asia and in Europe [135]. A VLP-based HBV vaccine for global use should offer protection against all HBV genotypes irrespective of where an individual lives. The same applies to HPV- and HEV-based vaccines. Although efforts have been made with the development of Gadarsil-9 to broaden the spectrum of protection against the oncogenic HPV types (HPV16, 18, 31, 33, 45, 52, and 58) prevalent in other parts of the world than those mentioned above, there are still some HPV types (HPV35, 39, 51, 59, etc.) associated with 10% of cervical cancer against which the vaccine does not protect (this is reviewed in [29]). For example, while Gadarsil-9 offers protection against the majority of HPV types associated with ~90% of cervical cancer cases in Africa, Europe, North America, and Latin America and the Caribbean, it only provides 86.5 and 87.5% protection in Australia and Asia, respectively (this is reviewed in [29]). The vaccine does not protect against HPV35, which is associated with 3.4% of cervical cancer cases in Africa, 1.6% of those in Asia, 2.3% of those in Latin America and the Caribbean, 1.8% of those in Australia, and 1.4% of those in Europe. HPV56 is another prevalent HPV type against which the vaccine does not protect, and it is associated with 1.4% of cervical cancer cases in both Europe and Africa and 1.0% of those in North America (this is reviewed in [29]). The development of a 14-valent candidate vaccine (including HPV35, 39, 51, 56, and 59, which are not covered by Gadarsail-9) will protect against the aforementioned HPV types worldwide.

To be effective globally, an HEV-based VLP vaccine such as Hecolin must provide protection against all four HEV genotypes (HEV1–4) prevalent in humans in different geographical regions around the world. As mentioned above, the Hecolin vaccine is derived from HEV genotype 1 and it cross-protects against genotypes 2 and 4. The vaccine is also expected to offer some degree of cross-protection to genotype 3 [41,42,136]. However, since genotype 3 is prevalent worldwide [137], while genotypes 1 and 2 are prevalent mainly in developing countries in Asia and Africa, where the vaccine is currently being tested (i.e., in Bangladesh, but not in Africa), it is crucial to conduct protective studies that include genotype 3 in other regions of the world to ensure the vaccine’s effectiveness on a global scale; genotype 4 is also prevalent in China. It is worth mentioning that the Hecolin vaccine is developed using the bacterial expression system. The use of the bacterial expression system is cheaper than the use of eukaryotic expression systems. However, bacterial expression systems, unlike eukaryotic expression systems, lack post-translational modifications such as glycosylation. Although the lack of glycosylation has been associated with the poor efficacy of some vaccines [138], this does not seem to be the case with the Hecolin vaccine; as a matter of fact, monkeys vaccinated with the vaccine were completely protected from infection with HEV genotype 1 [41].

As mentioned earlier, VLPs (especially those from HBV and HPV) have also been used as display platforms to enhance the immunogenicity of peptides derived from other infectious agents (e.g., bacteria and other viruses) and non-infectious agents (e.g., cancer) [46,49,80,81,82,139], with the ultimate goal of protecting against these agents. However, using VLPs from human viruses as display platforms for foreign peptides presents a challenge due to the presence of pre-existing antibodies in the human population, either from natural infection or vaccination, against the platforms (this is reviewed in [140]). In fact, some studies have shown that high levels of pre-existing antibodies to some platforms can reduce the immunogenicity of the platforms as well as the efficacy of heterologous antigens displayed by the platforms [141,142,143,144,145,146]. For example, pre-existing maternal antibodies to poliovirus in children have been shown to reduce vaccine efficacy by up to 28% following the immunization of the children with the same antigen [144]. To overcome this limitation, VLPs from animal viruses (which do not colonize humans), plant viruses, and bacterial viruses can be used to display foreign peptides.

Overall, VLPs are versatile and efficient platforms for vaccine development. They can be used to develop vaccines which act not only against the viruses from which they are derived, but also against other infectious and non-infectious agents. Furthermore, they are excellent platforms for the development of hybrid/mosaic vaccines against viruses with segmented genomes, especially those that are transmitted by insects (e.g., AHSV). Their inability to replicate in insects reduces the risk of reassortment events that can occur with live-attenuated vaccines developed from viruses with segmented genomes. Additionally, a VLP-based vaccine for viruses such as AHSV may also help in differentiating vaccinated horses from infected horses, which may be challenging with a live-attenuated vaccine.

## Figures and Tables

**Table 2:** VLPs derived from human viruses (chimeric VLPs) displaying foreign antigens.

Name of Virus Used to Make VLPs	Structural Protein Used to Make VLPs	Capsid or Envelope Proteins	Foreign Antigen Displayed on VLP	Expression System	Immune Responses	References
Parvovirus B19	VP2	Capsid	Linear epitopes from human herpes simplex virus (HSV type 1) and mouse hepatitis virus (MHV)A59	*Sf* insect cells	Mice immunized with the chimeric VLPs were partially protected against infection with HSV or MHV.	[79]
* HPV	Major capsid protein	Capsid	HPV16 L2 (aa 17–36)	*Sf*9 insect cells	Sera from immunized mice neutralized or protected against infection with HPV pseudovirus types: 5/6/11/16/18//26/31/33/34/35/39/43/44/45/51/52/53/56/58/59/66/68/70/73	[80,81,82]
HBV	HBsAgS	Envelope	130-amino acid from the C-terminus of KatA of Helicobacter pylori	Human hematoma-7 cells	Bacterial load was reduced in mice vaccinated with chimeric VLPs by at least 50%	[46]
			HCV envelope glycoprotein 2 epitopes (HCV 412–425, 434–446, 502–520, and 523–535)	*Leishmania tarentolae*	Sera from mice immunized with VLPs displaying epitope 412–425 neutralized (80–100%) HCV genotypes 1a, 1b, 4a, and 5a	[47]
Norovirus	Protrusion domain	Capsid	10 different epitopes from four capsid proteins (VP1-VP4) of enterovirus A (EV71)	Rosetta competent cells (DE3 strain)	Mice immunized with VLPs displaying amino acid 176–190 from VP3 and amino acid 208–222 from VP1 (both from EV71) were completely protected from E71 infection.	[83]
			Rotavirus VP8 (159 amino acid from the capsid)	*E. coli* BL21 cells	Immunized mice reduced mouse rotavirus shedding by 89–99.2%.	[84]
HIV-1	VHIV-1 Nef ^mut^	Envelope	HPV16 E7 protein	HEK 293 G protein-coupled receptor cells	Mice immunized with VLPs developed anti-E7 cytotoxic T-cell response and were protected from developing HPV-related tumors.	[85]
HBV	HBcAg	Capsid	Peptide from HPV 16 E7 protein (amino acid 49–57)	*E. coli*	VLPs suppressed the development of tumors in a TC-1 grafted mice model.	[49]
HBV	HBsAg	Envelope	Dengue virus envelope protein (specifically, domain III of dengue virus 1–4)	*P. pastoris*	Mosaic VLPs elicited neutralizing antibodies against dengue 1–4. Antibodies protected AG129 mice against lethal challenge with dengue virus 4	[48]

* Received approval for phase I clinical trial.

**Table 4:** VLPs derived from animal viruses (chimeric VLPs) displaying foreign antigens.

Name of Virus Used to Develop VLPs	Structural Antigen Used to Make VLPs	Capsid or Envelope Proteins	Foreign Antigen Displayed on VLP	Expression System	Immune Responses	References
Infectious bursal disease virus	VP2 precursor (466-residue)	Capsid	HA and M2 protein epitopes derived from the mouse-adapted A/PR/8/34 influenza virus	*Trichoplusia ni* (H5) insect cells	A total of 100% of mice immunized with chimeric VLPs were protected against lethal challenge with influenza virus; in control groups; 83% of mice died 7–9 days after infection.	[106]
Rabbit hemorrhagic disease virus	VP60	Capsid	T-helper epitope from 3A protein of foot-and-mouth disease virus	*Trichoplusia ni* (H5) insect cells	Vaccinated pigs generated specific IgA and IgG responses, had high IFN-γ-secreting cells and 3A-specific lymphoproliferative specific T cell responses.	[107]
Influenza A virus	HA, NA, and M1 of H5N1 virus	Envelope	Ectodomain of Newcastle disease virus hemagglutinin-neuraminidase protein	*Sf*9 insect cells	Vaccinated chickens were completely protected against Newcastle disease F48E9 virus.	[108]
Canine parvovirus	VP2	Capsid	Receptor binding domain of MERS-CoV	*Sf*9 insect cells	Sera from mice immunized with VLPs neutralized a pseudo-MERS-CoV. A balanced T-cell (Th1 and Th2) response was elicited.	[94]
Newcastle disease virus	M protein	Envelope	Brucella antigen BCSP31	*Sf*9 insect cells	Mice immunized with VLPs elicited humoral and cellular immune responses in mice. Protection efficacy against a virulent strain of *Brucella melitensis* (strain 16M) was comparable to a commercial live-attenuated vaccine: *Brucella melitensis* strain M5.	[95]
PCV-2	Capsid protein	Capsid	Ectodomain of matrix protein 2 (M2e) of influenza A virus	*E. coli*	VLP-immunized mice were protected against challenge by A/swine/Zhucheng/90/2014 (H1N1) or A/swine/Henan/1/2010 (H3N2) strains. Control mice died from the challenge. Immunized mice were also protected against human [A/Puerto Rico/8/1934 (H1N1)] and avian [A/chicken/Guangzhou/GZ/2005 (H9N2)] influenza viruses.	[109]

**Table 6:** VLPs derived from bacterial viruses (chimeric VLPs) displaying foreign antigens.

Name of Virus Used to Develop VLPs	Structural Antigen Used to Make VLPs	Capsid or Envelope Proteins	Foreign Antigen Displayed on VLP	Expression System	Immune Responses	References
Bacteriophage AP205	Capsid protein	Capsid	West Nile virus envelope protein domain III (WNV EDIII)	*Nicotiana benthamiana*	Mice immunized with only 5 μg of chimeric VLPs elicited potent IgG responses (1:32,000) that were 4-fold higher compared to mice with immunization with only soluble WNV-EDIII protein.	[126]
Bacteriophage MS2	Capsid protein	Capsid	Concatemer of L2 peptides from two HPV types and a consensus sequence from different HPV types	*E. coli*	Mixed MS2-L2 VLPs protected mice against 11 oncogenic HPV pseudovirus types which are associated with around 95% of cervical cancer. Spray-freeze drying increased the thermostability of the VLPs (stored at room temperature for up to 60 days).	[116,117]
Bacteriophage Qβ	Capsid protein	Capsid	Microtubule-associated protein tau peptide	*E. coli*	Mice immunized with chimeric VLPs had reduced levels of hyperphosphorylated pathological tau.	[127]
Bacteriophage P22	Capsid protein	Capsid	B and T epitopes (OVA_B_ and OVA_T_ peptide) of ovalbumin	*E. coli*	VLP-OVA_T_ vaccine significantly inhibited tumor growth and lowered the proportion of myeloid-derived suppressor cells among tumor-infiltrating lymphocytes and splenocytes.	[119]
Bacteriophage PP7	Capsid protein	Capsid	L2 peptides from eight different HPV types	*E. coli*	Mice immunized with VLPs (individually or as a mixture) elicited high-titer anti-L2 IgG serum antibodies; immunized mice were protected from a high-dose challenge with HPV pseudoviruses (PsVs).	[118]
